# Editorial: Inflammatory pathways in cardiometabolic diseases: mechanisms, biomarkers, and therapeutic insights

**DOI:** 10.3389/fcvm.2025.1737584

**Published:** 2025-12-04

**Authors:** Lei Wang, Yue Liu

**Affiliations:** 1 Chinese Medicine Guangdong Laboratory, Guangdong Hengqin, China; 2 Department of Emergency Medicine, Dongguan Hospital of Guangzhou University of Chinese Medicine (Dongguan Hospital of Traditional Chinese Medicine), Dongguan, China; 3 Xiyuan Hospital of the Chinese Academy of Traditional Chinese Medicine, Beijing, China

**Keywords:** cardiometabolic diseases, inflammatory microenvironment, immunometabolism, biomarkers, therapeutic targeting

The inflammatory microenvironment, composed of immune cells, extracellular matrix, growth factors, and inflammatory mediators, plays a central role in the pathogenesis and progression of cardiometabolic diseases (CMD) Liu et al., (1). Although its contribution to immune cell infiltration, endothelial dysfunction, oxidative stress, and metabolic dysregulation is well recognized, significant knowledge gaps persist regarding the specific molecular mechanisms, biomarkers, and therapeutic opportunities (2, 3).

This Research Topic seeks to address these gaps by compiling cutting-edge research on the inflammatory microenvironment in CMD, with the aim of elucidating underlying biological mechanisms, advancing biomarker development, and evaluating the efficacy of anti-inflammatory therapies, thereby fostering novel strategies for disease management.

The understanding of the inflammatory core in cardiometabolic diseases (CMD) has shifted from the established paradigm of systemic, low-grade inflammation to a targeted exploration of tissue-specific immunometabolic crosstalk (4). In their review, Al-Shahrabi et al. compellingly demonstrate that disruptions in amino acid metabolism and mitochondrial redox balance are not merely secondary phenomena but active drivers of pathological cardiac remodeling. This theme of metabolic-immune crosstalk is further elaborated by Liu et al., which meticulously delineates how diverse insults—from oxidized lipids to hyperglycemia—converge to orchestrate a pro-inflammatory niche across various CMDs. Long considered merely a glycolytic byproduct, lactate is now recognized as a key regulator of diverse physiological and pathological processes (5). Exemplifying this expanded role, the work by Zhang et al. introduces lactylation as a previously underappreciated mechanism, redefining lactate from a metabolic waste product to a critical immunological messenger that directly couples glycolytic flux to epigenetic reprogramming and inflammatory gene expression. The works in this Topic collectively demonstrate that metabolic signals not only accompany but actively orchestrate innate immune memory and sustain maladaptive inflammatory responses across cardiovascular and metabolic tissues.

A principal challenge in the field lies in translating mechanistic insights into clinically applicable tools. This Topic highlights significant progress in the domains of biomarker discovery and risk stratification, reflecting a shift from reliance on single molecules toward integrated, pathophysiologically grounded indices. The study by Meng et al. exemplifies this trend by validating the monocyte-to-high-density lipoprotein cholesterol ratio (MHR)—a metric that concurrently reflects inflammatory cellular activity and dyslipidemia—as a marker of obstructive sleep apnea severity. Similarly, Liu et al. demonstrate that the epicardial adipose tissue mass index (EAMI), derived from routine imaging, provides superior prognostic value compared to conventional risk scores, thereby quantifying the risk attributable to a key paracrine inflammatory organ. Further reinforcing this narrative, the contributions of Sun et al. and Wang et al. underscore the prognostic significance of metabolic byproducts such as the blood urea nitrogen-to-albumin ratio and serum uric acid, highlighting an unequivocal link between metabolic waste accumulation, inflammation, and clinical deterioration.

Ultimately, the delineation of pathogenic pathways must inform therapeutic innovation. The articles presented here offer insightful perspectives on future treatment strategies, spanning from the network-modulating properties of traditional medicine to the precision of novel molecular targets. Du et al. provide a rigorous mechanistic foundation for Shexiang Tongxin Dropping Pills, illustrating its synergistic efficacy against both oxidative stress and the potent TNF-α/IL-6 inflammator*y* axis. Concurrently, the metabolomic study by Wang et al. identifies dysregulated bile acid metabolism as a specific pathogenic driver, thereby unveiling a potential new avenue for intervention. Looking forward, the comprehensive review by Chen et al. builds a compelling argument for vascular adhesion protein-1 as a druggable target, whose inhibition may directly attenuate vascular inflammation.

In conclusion, the studies featured in this Research Topic converge on a clear message: inflammation in cardiometabolic diseases is not a static phenomenon but a dynamic, networked process deeply embedded within metabolic regulation. It functions simultaneously as cause and consequence, signal and effector. Together, these findings urge the scientific community to embrace a more integrative framework—merging metabolic and immunologic insights—to develop biomarkers and therapeutic strategies that capture the complex pathophysiology of CMD. We are confident that the foundational work collected here will inspire future research and accelerate the translation of these insights into more precise and effective clinical interventions for patients with cardiometabolic disease.
